# The ethics of interrogation and the American Psychological Association: A critique of policy and process

**DOI:** 10.1186/1747-5341-3-3

**Published:** 2008-01-29

**Authors:** Brad Olson, Stephen Soldz, Martha Davis

**Affiliations:** 1 Human Development and Social Policy, Northwestern University, 2120 Campus Drive, Evanston, Illinois 60208, USA; 2 Boston Graduate School of Psychoanalysis, 1581 Beacon St., Brookline, Massachusetts 02446, USA; 3 John Jay College of Criminal Justice, City University of New York, 899 Tenth Avenue, New York, New York 10019, USA

## Abstract

The Psychological Ethics and National Security (PENS) task force was assembled by the American Psychological Association (APA) to guide policy on the role of psychologists in interrogations at foreign detention centers for the purpose of U.S. national security. The task force met briefly in 2005, and its report was quickly accepted by the APA Board of Directors and deemed consistent with the APA Ethics Code by the APA Ethics Committee. This rapid acceptance was unusual for a number of reasons but primarily because of the APA's long-standing tradition of taking great care in developing ethical policies that protected anyone who might be impacted by the work of psychologists. Many psychological and non-governmental organizations (NGOs), as well as reputable journalists, believed the risk of harm associated with psychologist participation in interrogations at these detention centers was not adequately addressed by the report. The present critique analyzes the assumptions of the PENS report and its interpretations of the APA Ethics Code. We demonstrate that it presents only one (and not particularly representative) side of a complex set of ethical issues. We conclude with a discussion of more appropriate psychological contributions to national security and world peace that better respect and preserve human rights.

## Introduction

The American Psychological Association (APA) has a long history of opposing the misuse of psychological knowledge in practice, assessment, research and any other activity utilizing the tools of the field [1,2]. The *APA Ethical Principles of Psychologists – Code of Conduct *has long been the guide to both acceptable and prohibited behavior [3,4], and has long ensured the proper and safe use of psychological methods. It protects U.S. psychologists, but most important of all, those who are most exposed and most vulnerable to the misuse of psychology and its tools [5].

In the wake of September 11, 2001 and the subsequent "war-on-terror", the APA decided to address the ethical implications of psychologist "contributions" to U.S. national security. Calling the situation an "emergency", APA President Ronald Levant authorized the APA Presidential Task Force on Psychological Ethics and National Security (PENS) [6]. The task force report and process generated a number of controversies. One set revolved around the appointment of task force members who were primarily psychologists serving active military or working in some then current capacity with the U.S. Department of Defense (DoD) [7]. Criticism of the task force composition grew when the identification of members revealed deep connections with intelligence gathering, detainee interrogations, and related operations within the DoD [8].

The report, hereafter referred to as the PENS Report, was completed in 2005 [6]. Shortly thereafter, the APA Board of Directors and the APA Ethics Committee accepted the report as policy. The APA leaders had not only underlined the importance of psychologists contributing to "national security", but that the profession had a responsibility to serve U.S. society [9]. APA rules and regulations on such task forces require "emergency" reports to be ratified by the Council of Representatives at the next Council meeting. This vote did not then occur and has never occurred. However, APA leadership have on several occasions claimed that Council approved the report, and on almost as many occasions, publicly retracted the statements. Most consequential, though, is that the APA Ethics Committee did officially approve the report, calling it, without a single alteration, consistent with the APA Code of Ethics. Yet it was the content of the report, and its seeming inconsistency with psychological ethics, that provoked the strongest feelings throughout the APA membership, and less than favorable reactions from the media, among them *The New York Times *[10].

Upon adoption, the PENS report became policy binding upon members of the APA involved in detainee interrogations and other U.S. national security activities. As psychologists and members of the APA, the authors will elucidate a number of controversial issues regarding the PENS process and weaknesses in the reasoning of the PENS report.

## The incompatible nature of the PENS report and the ethics code

It would be inaccurate to say there was nothing positive in the PENS report. It had, after all, reaffirmed the 1982 APA Resolution against *Torture and Other Cruel, Inhuman, or Degrading Treatment*. Also, in 2006 and again in 2007, the Council of Representatives supported further resolutions against torture with ever tighter and more explicit prohibitions [11,12]. However, each resolution had its own problems and serious omissions and each resolution has preserved the direct role of psychologists in military and intelligence interrogations. Each resolution had also stopped far short of a "bright-line" position that, like the American Psychiatric Association and American Medical Association, would prohibit the direct involvement of members in these interrogations [13].

Further supporting PENS reasoning, the APA Council of Representatives in August of 2007 soundly defeated an amendment attempting to restrict psychologists to health-related services in settings that deprived persons of basic human rights. In essence, the APA never swayed from PENS Report Statement #7, concluding that "psychologists may serve in various national security-related roles, such as a consultant to an interrogation..." [[6], p. 6]. It is true the Council of Representatives never voted to approve the PENS report in its entirety, yet while Council has expressed reservations and at one point demanded a certain set of actions, they have never fully opposed the policy. Controversies regarding the PENS Report and interrogation policy among Council members and the APA membership have nevertheless continued unabated.

The recommendations of the PENS Report, its basic premises, operational definitions, and reinterpretations of the ethics code have long warranted a detailed evaluation. This is the goal of our critique. We hope to develop ethical considerations regarding psychologists and detainee interrogations, and to help work toward improved policies on the role of psychologists within these settings. We believe that this critique supports an absolute prohibition of psychologists' direct involvement in these interrogations, and that this is the safest, wisest and most ethical course of action for the American Psychological Association and its members. Such a ban, we believe, may also be the safest and surest course for national security [13].

## All U.S. psychologists accountable to all sections of the code

The PENS Report, in one of its stronger statements, explicitly affirmed that all U.S. psychologists, regardless of their different applied, research-based, or practitioner roles, were to be held fully accountable to *all sections *of the APA ethics code [[6], p. 1]. This is a key conclusion, insisting, for instance, that a "clinically-trained" psychologists acting in a "consultant role" is responsible to both *clinical *and *consulting *sections of the code, and even those sections applying to *assessment *and *research*. It equally reaffirms the task force's commitment to existing ethical principles and standards within the code, supported also, of course, by the approval of the APA Ethics Committee^a^. To a large extent, we also feel comfortable with the current Ethics Code (with the important exception of new 2002 clauses, such as 1.02, that allow compliance with local law, regulations, or governing authorities when institutional demands conflict with the psychologist's ethical responsibilities). However, our interpretations of the Ethics Code and its relationship to PENS differ fundamentally from the Ethics Committee's position. In fact, we reach the opposite conclusion, namely that the ethics code demonstrates, in our interpretation, that it is fundamentally *unethical *for psychologists to directly participate in military and intelligence interrogations.

## A "New" ethical dilemma and the national security frame

The PENS Report stated that war-on-terror interrogations involving psychologists produced a completely new ethical dilemma, never before encountered by the APA [10]. Given psychologists' long-established place in military and CIA intelligence gathering, and given the vast precautions suggested by the literature on social influence (see discussion of Milgram below), there was little that should have been considered new. What was a first was the APA's vigorous and official endorsement of psychologist involvement under the banner of "national security" interests. The power of this banner – whether it was created unconsciously and took on a life of its own or whether it was purposefully designed to influence others – should not be underestimated.

## Unresolved conflicts within the task force

Many of the problems with the task force report can be understood through the biased majority influence that led to several rather questionable (and objectively ambiguous) conclusions. Its contradictions were at least partially a product of several core issues that arose either from majority or minority members of the task force. In the report, several areas of disagreement between task force members were documented.

One primary conflict concerned whether the content of the task force proceedings would be open and public or whether they would remain confidential. The majority, the seven of nine voting task force members, insisted that the entire discussion and decision-making process remain secret. This decision continues to reverberate, casting the deliberations in a negative light.

Another conflict among the task force concerned whether the standards and definitions of torture would be based solely on current U.S. administration standards or on international guidelines, best represented by the Geneva Conventions, the U.N. Declaration of Human Rights, and the Convention against Torture. The majority members of the task force once again, with the help of the APA leadership present, favored using exclusively U.S. over international definitions, and this standard is evident throughout the report's final conclusions.

## Theoretical abstractions

The PENS report also suffers from contradictions and ambiguities that may be largely attributable to its remaining on a plane of theoretical abstraction. Evident throughout is a complete absence of concrete examples that might have brought further light for the task force on the ethical challenges that psychologist-guided interrogations really entail. We know now that these excessive abstractions are partially due to restrictions imposed by the APA leadership and enforced at the meeting [14]. The leadership persistently refused task force members any latitude to consider reports on unethical and unlawful interrogations that had been widely reported by human rights groups and the media throughout the first half of 2005.

The PENS Report, for instance, never mentions the well-substantiated reports of detainee abuse. It never cites the numerous reports of psychologist involvement in these abuses, or even hypothetical dilemmas that psychologists might encounter. In fact, throughout the PENS Report, no US detention center – Guantanamo, Abu Ghraib, nor the CIA's black sites – is ever mentioned. This denial of information is consistent with APA leadership communications well beyond PENS. Up through August of 2007, the APA has never officially acknowledged any wrongdoing by any psychologist, even though the evidence has been indisputable, some of which will be described here. Serious debate requires that the participants have the autonomy to ponder real-world examples, as well as best and worst case scenarios. When documented events are intentionally ignored, group process is inevitably challenged in reaching ethical and reality-based conclusions.

Despite the directives of the APA leadership to avoid consideration of well-documented abuses, sections of the PENS Report acknowledged that "...because of a setting's unique characteristics, an individual [i.e., the detainee] may not be fully able to assert relevant rights and interests" [[6], p. 7]. However glancing and understated this statement seems, such references to the blatant violations of detainee rights are in stark contrast to all other sections of the document that dwell on the patriotic abstractions of "national security."

While much was known about psychologist involvement in detainee abuse prior to the PENS report, what has become progressively clearer is that the methods used by interrogators, guided by Behavioral Science Consultant Teams (BSCTs) [15-17], have been intentionally shaped by psychologists [18-20]. Many of the most objectionable interrogation strategies had been re-designed by psychologists from U.S. military programs, primarily the *Survival, Evasion, Resistance, Escape *(SERE) program, originally intended to protect U.S. soldiers from undesirable thought reform [21,17,23].

According to international definitions, and the understanding of the SERE program itself, SERE-based interrogation procedures constitute torture [22,23]. Official reports and numerous journalists over the last several years have provided extensive documentation depicting how these SERE techniques were used in U.S. interrogation practices by SERE-trained psychologists, both in DOD and CIA detention facilities [24,25,21,22]. Yet, however despicable, psychology should never let these "enhanced techniques" cause us to ignore the only somewhat more subtle techniques prescribed in the Army Field Manual [26], the common guide for all U.S. military interrogations. In the Army Field Manual, allowable interrogation tactics include deception, fear escalation, ego harm, isolation, and psychological disorientation. Regardless of whether these techniques are ethical for professional interrogators, they are morally problematic for psychologists, given the clearly circumscribed ethical underpinnings of the profession.

## The proposed casebook

To a degree, the PENS Task Force recognized the report's abstract nature. It was proposed that the task force would continue working after the publication of the report on a casebook that would provide specific examples of ethical dilemmas and concrete instances of appropriate psychologist behaviors [27]. Despite this written commitment, and a call in 2004 by the APA Council of Representatives for the casebook's completion [28], work on it only commenced after the 2007 Convention. The 2007 APA resolution [12] reaffirmed the APA's commitment to a casebook and commentary, this time in the hands of the Ethics Committee. A call has been published requesting member recommendations for the casebook, but those recommendations are not due until March of 2008 [27]. While little relief is in sight for detainees brought to Guantanamo in 2002, or those disappeared into the CIA's secret detention facilities, the rate of progress to date suggests the casebook will not be developed and made policy until the next U.S. presidential election is upon us, or possibly even the next administration. This speed and style is in dire contrast to the initial "emergency" call for the PENS Task Force, and the quick execution of the original report.

## Operational definitions of interrogation and torture

It is worth examining other sources of ambiguity in the PENS Report. This includes an absence of operational definitions presented for the most critical interrogation/torture-related terms. The PENS Report was said to be a reaffirmation of the APA's earlier *Resolution Against Torture*, yet the report never defined when interrogation becomes torture. The 2006 APA Resolution against Torture [11] cites broader human rights definitions, yet what the APA actually considers an "ethical" interrogation and what it constitutes as torture was never spelled out with any precision in the PENS report. Sound, ethical decisions require sound reasoning and a consistently agreed upon language.

Some values of the psychological profession were never addressed in the PENS report, such as the ethics code's prohibition on *coercing *participants into research. What is *coercion *in research or even in Guantanamo interrogations? Detainees are incarcerated indefinitely without adequate legal representation. If they provide interrogators with useful information, they are rewarded with less isolation and other amenities. If they do not cooperate, or have no useful intelligence, they are well aware they will be punished further. Quite apart from allegations of outright torture and abuse, these conditions make the interrogations *by definition *coerced.

One PENS term most in need of an effective definition is "degradation." Holding off on "cruel" and "inhuman" for the moment, the report prohibits psychologists from engaging in "degrading" behaviors (p. 1). Even if psychologists were experts in human rights law, they are left with little APA guidance on the term's meaning in practice. Strict psychological criteria are needed to accompany such APA statements. The definitions provided by the American Heritage Dictionary are a start. The verb "to degrade" is in part:

"1. To reduce in grade, rank, or status; demote. 2. To lower in dignity; dishonor or disgrace. 3. To lower in moral or intellectual character; debase. 4. To reduce in worth or value. 5. To impair in physical structure of function."

As argued by Mayer [16], "only in an Orwellian world could the actions of behavioral scientists in these settings be termed anything but a process of 'degradation'." Degradation is an ongoing, psychologist-designed experience for detainees. As stated, even outside the "cruel" and "inhuman" *enhanced techniques *characteristic of CIA interrogations, the basic Army Field Manual [26], again the guidebook for interrogations, proffers strategies of *fear up harsh *and *ego down*. Such techniques are indisputably degrading, and antithetical to the tenets of any mental health profession.

## A real case study

To push through the abstractions and concretize our understandings of degrading practice, there is fortunately one explicit record on a BSCT psychologist and his directorial function in the interrogation process. This leaked interrogation log [29] included a Major L., whose identity was publicly revealed to be Dr. John Leso, a counseling psychologist and APA member [19]. As a BSCT psychologist, Dr. Leso consulted on the interrogation of Mohammed al-Qahtani. By any reasonable definition, the interrogation was extreme torture [30-33]. It was not clear from the interrogation log that Dr. Leso was present at each and every stage of the interrogation process, and his name tends not to appear in sections documenting the most extreme tactics. Nevertheless, the log documents Leso's presence during an ongoing interrogation, often crossing the border into torture.

Those clearly documented behaviors of Dr. Leso might be considered comparatively mild; although, they are revealing in the light of the ethics code and what is known about BSCT practice. Dr. Leso, for instance, asked the interrogators to play cards in front of Mr. al-Qahtani to determine whether Mr. al-Qahtani was moving toward a psychological identification (i.e., dependence) on the interrogators. If Mr. al-Qahtani was seeking the interrogators' attention, according to Dr. Leso's sophomoric psychological reasoning, this would confirm the interrogation was progressing as planned. Dr. Leso called for the detainee to be kept awake, and to be placed in a swivel chair, moving him in any direction according to the will of the interrogator(s). This rolling and swiveling mechanism was based upon Dr. Leso's belief that Mr. al-Qahtani was relying on a coping technique consisting of the detainee focusing his attention on a single point on the wall. Dr. Leso therefore directed the interrogators to disrupt the detainee's ability to hold his attention. The swivel chair served this purpose. This combination of *dependence *and *disorientation *is indeed degrading^b^.

We wish there was more known about Dr. Leso's involvement. We wish we knew what part, if any, Dr. Leso played in the more extreme forms of torture to which Mr. al-Qahtani was subjected. We know that Dr. Leso was potentially involved in abusive techniques that likely contributed to harm. Regardless of what else Leso did, we have trouble imagining – as a professional psychologist and as an observant human being – that he didn't notice signs of psychological and physical deterioration evident in Mr. al-Qahtani expression and in his physical behavior^c ^[32]. What was Dr. Leso's responsibility to protect Mr. al-Qahtani's mental health? Whatever it was, Dr. Leso's coordination of the interrogation only exacerbated harm.

The case of Dr. Leso and Mr. al-Qahtani speaks to the reality of psychologist involvement in interrogations. It is our belief that complex ethical considerations are involved in any instance where psychologists participate in the overriding of the wishes of human beings. These ethical calculations become even more complex in situations where psychologists are dealing with humans refusing to utter a single word, under conditions lacking virtually all legal protections and without an attorney or advocate present. There is no evidence that the PENS task force or the ethics committee considered these complex moral issues.

We must also protect ourselves from falling into the pseudoscience trappings recurrent throughout our discipline's history [34], regardless of ego-based and careerist temptations. Psychologists, like others, often cannot tell when a detainee is reluctant to provide information or when they simply have no information to give [35]. Subtle techniques of verbal pressure, it must be reiterated, are as ethically problematic for psychologists as water-boarding, the administration of drugs, or the sexual or religious torments practiced in these war-on-terror detention centers [36-39]. Because the risk of coercion and torture is inherent in these settings, the bright-line position of no psychologist participation in the interrogation process, at least for the time being, takes precedence over our best but highly unrealistic wishes that psychologists are there to minimize harm [13,40].

## The utilitarian fallacy of national security

The principles and standards of the APA Ethics Code apply first and foremost to the protection of those individuals or groups most directly impacted by the psychologist's professionally-related engagement in the world. The ethics code does, in at least one instance, emphasize larger societal goals that can take many forms. For the PENS tasks force, the national security goal, a largely societal emphasis, is the cornerstone of its recommendations (p. 2) see also [41] for a good discussion on the excessive attention in psychological ethics on the distal, societal benefits to the neglect of proximal, individual benefits).

The societal "national security argument" in the PENS report is that the potential distal protection of Americans supersedes the psychologist's responsibility to the most immediately vulnerable individual (i.e., the detainee) (p. 2). This is in every sense a utilitarian argument, suggesting that risking a certain degree of harm, whether mental or physical, to an individual (really many individuals over an extended period) is acceptable to reduce the possibility that many others will be harmed at a future date (see also [9] for a further explication of this argument).

We are skeptical of this particular assessment, assuming even that its basic foundations are reasonable. What is more, these utilitarian arguments rarely incorporate "all societies" within the formula; instead, the judgments historically tend to be biased to protect the proponent's own in-group, most often to the detriment of all others, i.e., the out-group [42].

In the PENS Report, the in-group utilitarian argument is defended through appeals to the ethics code. In particular, *Principle B: Fidelity and Responsibility *is used and its tenet that psychologists should be "aware of their professional and scientific responsibilities to society" [[4], p. 3]. Fidelity and Responsibility here is extrapolated by the PENS Report to "gathering information that can be used in our nation's and other nation's defense." (p. 2). We believe this application of Principle B is particularly idiosyncratic and over-reaching. There are other psychologist "responsibilities to society" in this context that tend to be more compelling, such as the responsibility to avoid participation in activities that could harm society's trust in the discipline. This explicitly utilitarian phrase, moreover, is only a small portion of Principle B, which more thoroughly asserts that psychologists should, "...seek to manage conflicts of interest that could lead to exploitation or harm" (p. 3). This clause, we believe, is far more apt to the present situation, characterizing the ethical dangers of psychologist-involvement in "war-on-terror" interrogations.

In general, any ranking of potential contributions to national security above protections of the most vulnerable human clients (and trust in psychologists as caregivers) is a high risk proposition for psychology as a profession [43]. This is not only an unacceptable ranking of priorities; it is putting highly speculative claims that can never be substantiated above the reality of abuses that have indisputably been witnessed across settings and over multiple occasions, spanning at this point over half a decade. There is no real way to confirm or disconfirm the utilitarian assessment. The very nature of these interrogations and the conditions of detention have, however, been widely and repeatedly denounced across much of the world [44,45]. And many expert political analysts have made sound arguments that they have placed everyone at higher risk for acts of terrorism. At a minimum, utilitarian arguments, to be valid, would need to encompass these unintended side effects of the government activities being aided by psychologists.

The truth is that APA interrogation policy has never adequately weighed the radiating international effects of detainee abuse, and their devastating impact on trust. With psychologists actively involved in detainee interrogations, reports of abuse are a threat to the whole profession. The damage to public trust may also be difficult to measure, but it is likely to be considerably more serious than any benefits believed to accrue from this policy. The utilitarian position is better framed as: what is best for the most people is what protects the dignity of the single individual, because the effects of endangering the single individual, to any degree, are far-reaching indeed [46].

## Domestic forensic psychology vs. foreign intelligence work: A false comparison

The battle over the APA endorsement of "psychologist-involved interrogations" continues a long, historical battle within psychology to preserve its reputation against the fear that members may attempt to psychologically control or obtain information from people against their will and outside their awareness [47]. Whether psychologists could ever successfully accomplish such feats remains an open question. Historically the profession has treated any effort by psychologists to control or extract information against a person's will, and in ways detrimental to their mental health, as problematic, if not definitively unethical.

In this regard, one phrase in the PENS Report – critical to its argument yet unsupported with documentation – is of particular concern. The report argues that "it is consistent with the APA Ethics Code for psychologists to serve in consultative roles to interrogation...*as psychologists have a long-standing tradition of doing in other law enforcement contexts*" (p. 1, italics added). This parallel between psychologists involved in foreign intelligence gathering operations and forensic psychologists working with domestic law enforcement is fundamental to the APA position supporting psychologist participation. There is a long history of U.S. forensic psychologists interviewing criminal suspects for dangerousness, fitness to stand trial, assessment of mental status and malingering, not to mention treatment of those who are incarcerated. U.S. psychologists conduct research on detecting deception, linguistic evidence of coercion in interrogations, and many other social psychological aspects of criminal investigations.

In its brief, precedent-implying phrase, the task force obscures three profound differences between the "long-standing traditions" of forensic psychology in U.S. "law enforcement contexts" and the context of psychologists in "war-on-terror" detainee interrogations. First, psychologists do not conduct interrogations in the U.S. and do not have authority over the law enforcement officers involved in them, as BSCTs have over the military interrogators they train and advise during interrogations [33] (see also log on Dr. Leso [29]). In the domestic criminal justice system, psychologists either offer training workshops on specific topics of value to investigators, or they present the results of linguistic or behavioral analyses of prior interviews, using methods developed that are too labor intensive for the investigators to master. They do not sit in on interrogations and help shape those events as members of the interrogation team. Any who do this in the U.S. are considered *former *psychologists, and now considered *law enforcement officers*, answerable to the chain of command of the police force or agency.

Second, and most important, research and consulting forensic psychologists work in settings where prisoners are afforded basic protections, including the right to an attorney, habeas corpus, and the right against self-incrimination. In contrast, psychologists aiding detainee interrogations are operating in systems in which violations of the rule of law are systemic and legal representation and other protections are denied. Such extreme violations of liberty are inevitably, in and of themselves, psychologically harmful and demeaning to the profession of psychology [48,49]. Despite the claim of precedent, information about domestic enforcement contexts bears little on whether psychologist involvement in detainee interrogations is adequately covered by the ethics code.

Third, military and CIA psychologists have far fewer independent outlets for advice and external support than mainland forensic psychologists. PENS Statement #12 asserts that "psychologists consult [with other psychologists] when they are facing difficult ethical dilemmas," and cites Ethical Standard 4.06 on Consultations (p. 8). Unfortunately, legally-binding secrecy, the chain of command, geographical separation, and other elements within the social climate of national security work can hamper the psychologist's ability to confer with objective, fellow psychologists outside, for instance, Guantanamo, a CIA black site, or a U.S. Navy Brig.

## The mixed-roles rule

A mainstay argument for prohibition against psychologist involvement is that the role inherently forces ethical compromises, and these constitute too great a risk for psychologists and detainees, not to mention the discipline of psychology and national security itself [13,40]. These compromises are evident in the PENS Report's "no mixed-roles" rule as anywhere. The no mixed roles rule is against "...mixing potentially inconsistent roles such as health care provider and consultant to an interrogation..." (p. 6). The intention is to make it more difficult for mental health information (e.g., on specific phobias, as in the classic novel *1984*) to so easily transfer from medical office to interrogation booth. The transfer is most easy when it is within one and the same person, although the practice of passing this information on through other modalities was common practice in Guantanamo [50,51]. Regardless of this PENS rule, national security interrogations take place in closed settings where the superordinate goal is to gather information. If it serves this goal, information can get passed from one professional to another [40,52,53].

The no mixed-roles rule also fails to recognize that licensed counseling and clinical psychologists are frequently used in these interrogation roles (as in the case of Dr. Leso). Such psychologists are very possibly used with the assumption that they can derive information about weaknesses or psychopathology from the statements, mannerisms, and other behaviors of the detainee. The no mixed-roles rule therefore seems to be little more than an inadequate, quick fix, which is simply an attempt to maintain psychologist involvement. The overlap of responsibilities within the same person is not the only source of role conflict detrimental to the detainee. The coexistence of psychologists as providers and interrogators, whether or not they are within the same person, produces ethical dilemmas in the form of *multiple relationships*, *dual-roles*, *dual loyalties*, and *conflicts of interest*. Despite any role demarcation, interrogated detainees are not likely to trust any professed distinction between care-giving and interrogating psychologists. Any semblance of trust a detainee might have had in a provider would inevitably erode with the practice of psychologists as interrogators (see also on dual relationship risks [54,48,55]. A report from the U.S. Army Surgeon General hints at the danger, stating that detainees have requested healthcare from BSCTs [56]. News travels, and that impacts the perception of psychology within detention centers and well outside the bounds of detention centers. Illusory firewalls will do little to protect vulnerable human beings from the misuse of psychology and the radiating effects of that mistreatment.

## The safety officer and behavioral drift

In order to clarify further the issue of multiple relationships, it is necessary to examine the "safety officer" and "behavioral drift" claims found in the PENS Report and other APA sources, all suggesting that psychologists make interrogations *safe *and *legal *[6,57-59]. One APA Monitor article implied that psychologists' involvement in interrogations had less to do with making interrogations "effective" and focused instead on maintaining the mental health of the interrogator, preventing what was called "behavioral drift". In other words, the psychologist's primary role is to prevent an interrogator's actions from escalating into torture [60]. It should not surprise anyone that interrogators or other professionals can lose their temper, experience moral lapses, or generally succumb to sadistic impulses [61].

Yet if preventing "behavioral drift" involves a psychologist providing mental health care to another interrogator, where is the separation of roles? How is *conflict of interest *avoided when a psychologist contributes to the gathering of intelligence from detainees while clinically monitoring the interrogator's behavioral drift? The psychologist cannot play the protector of a detainee while concurrently coaxing information from that same detainee. The psychologist cannot guide an interrogator on manipulation while working to monitor and restrain that same interrogator. What about potential conflicts of interest between interrogators, BSCT psychologists, and the chain of command? Any interrogation psychologist role in preventing "behavioral drift" involves, by definition, multiple and conflicting relationships. Meanwhile, who is monitoring the behavioral drift of the psychologist?

The APA argues that psychologists are uniquely qualified for the role of "safety officer." Why they, better than others, should be able to detect an interrogator's mounting frustration and progression to abuse compared to an army officer who was trained as a lawyer, linguist, anthropologist or school teacher is not explained. The term "behavioral drift" is used as if it were a subtle process that psychologists, with their clinical acumen, would be uniquely able to monitor. *Behavioral drift *is simply a euphemistic expression that obscures reality. It gives a pseudo-professional frame to what anyone could observe: the interrogator, through frustration, anger, and subsequent aggression, is beginning to abuse the detainee.

If psychologists are to prevent behavioral drift, ethical practice would require their total removal from the information-gathering context (see [61] for more on ethical drift interventions). Such psychologists could just as easily be independent from the chain of command, and have no role in monitoring or providing services to detainees. Psychologists could also train anyone who enters the interrogation booth against behavioral drift, and perform that training from a comfortable geographical distance.^d^

A more fruitful way to protect detainees lies in studying how to facilitate whistle-blowing – a concept in need of a less stigmatizing label. The term refers to raising concerns outside the chain of command, and, if needed, throughout the public sphere [62]. In these detention centers, there are interpreters, NGO observers independent of the chain of command, and military psychologists, all privy to acts of abuse. There is no evidence that practicing psychologists have any special qualifications for whistle-blowing, or are any more likely to do so than others. The empirical record produces only one potential case, that of Michael Gelles in Guantanamo. Unlike most potential whistle-blowers, Gelles was reporting abuse occurring in a chain of command other than his own. Perhaps more importantly, he was supported by his own command [63,64,40]. It was nevertheless a brave act, and one that needs to be more frequently emulated.

## Beneficence and non-maleficence

As pointed out above, the PENS Report is skewed in the direction of supporting psychologist involvement. It presents one side of an argument where clear pros and cons exist. The report selectively chooses sections of the ethics code supporting its recommendations and omits more salient standards that would oppose the task force's majority position. A number of APA documents assert that the PENS Report and the APA position on interrogation are based on Principle A of the Ethics Code [6,65]. While "do no harm" tends to be the half promoted by the APA, there are actually two sides to Principal A: Beneficence, or essentially "do good," which is a world of difference from the other side, non-maleficence, or "do no harm" [41]. Wisely, the creators of the APA Ethics Code made beneficence the first part of its first principle, symbolically preceding non-maleficence. According to the principle of beneficence, "Psychologists strive to benefit those with whom they work..." [[4], p. 3]. In various conversations with psychologists, there has been a tendency to regard the principle of beneficence as somehow quaint; too ideal for actual practice. Others have described Principle A as "a high level of abstraction, up with the moon and stars" and "lofty" [66]. Others have rejected any taking of the principle seriously, pointing to silly counterexamples; stating that punishing a child or giving a student a bad grade or producing therapeutic transference in a client could all be considered acceptable examples of "doing harm", and contrary to naïve notions of "beneficence." Such straw person arguments ignore that each of these examples possess the ultimate goal of bringing about benefits to the individual targeted by the intervention, an aim which is wholly absent in interrogation work on a detainee.

The PENS Report attributes *beneficence *to war-on-terror interrogations in two highly suspect ways. First, psychologist involvement in intelligence gathering and detainee interrogations is a positive good, the report claims, because it contributes to national security. In this framework the psychologist conceptualizes the "client" as the military, or U.S. society as a whole (p. 2)(see also [9]). The APA Ethics Code is based, in contrast, primarily on the individual or group receiving the psychological service, research attention, or consultation [4]. To make the "client" the U.S. military, intelligence, or U.S. society, is to make a radical shift in the focus of the ethics code, too radical to be considered a traditional or widely accepted interpretation. If every time "client" was mentioned in the code, it could be replaced by "the military", beneficence would be, "strive to benefit the military." As one moves through the ethics codes, the examples only become more absurd all the way into "Psychologists do not have sexual intimacies with clients". There are many cases where industrial/organizational psychologists are serving corporation interests and not the consumer per se. But these relationships are rarely as direct and potentially harmful as psychologists attempting to get information from unwilling detainees.

Another distortion of "beneficence" is the misconception that it primarily refers to psychologists in the "helping professions," and not to "applied/consulting psychologists" whose clients are institutions. Again, the PENS Report correctly asserts that all sections of the ethics code holds for all U.S. psychologists, whether providers, applied consultants, or researchers. Until beneficence is removed from the APA ethics code – which would be a mournful day for the profession – both halves of Principle A should be taken seriously and applied comprehensively.

## Other principles as commentary on beneficence

The APA Ethics Code in general is replete with fundamental principles, as shown here, that are avoided in the PENS Report, and that the APA leadership fails to discuss in its defense of its interrogation policy. Chapters could be written about each of a dozen principles and standards in the code and their incongruence with psychologist involvement. For example, *Principle C: Integrity, Honesty and Truthfulness *(p. 3) is relevant to the extensive deception that often takes place in war-on-terror interrogations. The majority of the original PENS task force, in opposition to integrity, honesty, and truthfulness, took a stand against the imperative psychological principles of informed consent and debriefing (p. 9).

*Principle D: Justice *(p. 3) must also be addressed. Some ethicists argue that a special relationship exists between the ethical principles of justice and beneficence (i.e., the wellness-justice nexus) [67]. In fact, reports from the American Bar Association and numerous human rights organizations about detainee treatment at Guantanamo Bay give more than adequate cause for a moratorium based on Principle D alone. The same can be said for *Principle E: Respect for People's Rights and Dignity *(p. 4) that emphasizes confidentiality, self-determination, and respect for culture and national origin [68]. Abusive religious and cultural techniques were once the bread and butter of war-on-terror interrogations.

## Do no harm: Principle and standard

"Do no harm" is one of the few aspirational principles in the code that is also an enforceable standard. Do no harm is the other side of the coin from beneficence; it has a protection function on the future actions of the psychologist or the psychologist's team; it is ostensibly the "safe" in the APA catch phrase, "safe, legal, and effective." It would also be akin to the "safety officer" role or in the prevention of "behavioral drift" except that the phrase *do no harm *is an absolute for psychologists' participation. In contrast to some other "ethicists", we do not believe the standard should be weakened with qualifications or continuums of harm [9]. When harm is treated as a legal set point, as in the interrogation safety officer role – when it is a degree of mental anguish that the psychologist decides should not go further – it places the psychologist in the insidious role of determining variations in the degree of harm. When harm is treated as a gradation to be gauged, when the psychologist is charged to judge how much harm, mental or physical – whether the harm is likely to be *temporary*, *permanent*, *prolonged*, *substantial*, or *significant *[12,69] – a dangerous door is opened up for the profession. A psychologist safety officer role is better off as part of the International Committee of the Red Cross that would strictly enforce among interrogators a "no harm" ethic. To the extent that the military desires a figure to judge gradations of harm, they are better off finding legal experts, who can better evaluate the procedures in the face of international law, without the illusion of a health professional's stamp of approval. Many critics have argued these decisions are outside the competence of psychologists. We agree. Competence is not a feasible goal when so many factors, including the protracted periods of detention, make predictions of future harm impossible. What is more important in our opinion, is that such competence is outside the psychologist's *ethical domain*, because it assails the absolute character of "do no harm."

## Standard 1.02

The most consequential and controversial flaw in the current ethics code is Standard 1.02 as it is written in the latest, 2002 version of the code [4]. In the *Introduction and Applicability *of the code, it is stated that should any conflict arise between a law and the ethics code, psychologists should take whatever steps they can to resolve the dilemma. If that fails, the code permits psychologists to follow the law, but, and this is critical, within the bounds of "human rights." Unfortunately, the mention of "human rights" is only in the *Introduction and Applicability *section. It is merely aspirational, not enforceable. The enforceable section, standard 1.02, on precisely the same topic, does not include language on *human rights*. Nor does it simply say that psychologists may follow the law over the ethics code. In addition to law, standard 1.02 includes the somewhat militaristic sounding "regulations" and "governing authority" as additional forces that permit a psychologist to abandon the ethics code. Moreover, unlike the Canadian ethics code for psychologists, 1.02 makes no mention of civil disobedience, nor does 1.02 provide guidance from the APA in resolving disputes based on moral conscience. Therefore, in rather plain language, the 2002 Code's enforceable 1.02 condones placing military commands and priorities over the ethical responsibilities of the psychologist.

The practical implications of 1.02 are evident in the case of Dr. Leso. Despite the objectionable nature of Dr. Leso's behaviors, he had been following the standard operating procedures (sop) of Guantanamo and of the Army Field Manual. "Regulations", the "governing authority", and U.S. administration interpretations of "law" all supported his actions, even if he violated enforceable standards such as *do not harass*, *do not harm*, and *do not exploit *[70]. We are aware of at least four ethics complaints against Dr. Leso filed with the APA Ethics office, dating back to at least August 2006^e^. Because standard 1.02 allows psychologists to treat these as superordinate to the code, it will be interesting to see what actions the APA ethics committee will take, if any, to hold Dr. Leso accountable.

In August of 2005, the Council of Representatives requested that the ethics committee address the loophole of 1.02. No changes in the ethics code have been made. The 2007 APA resolution against torture, to its credit, did include language that stated orders could not excuse torture or cruel, inhuman, and degrading treatment; although, whether this resolution truly supersedes Ethics Code provision 1.02 and whether actions like Dr. Leso's fall under the APA's definition of "degrading" treatment, remains unclear^f^. Further, the 2007 APA Resolution only addresses conflicts between laws/regulations and the injunction against participating in *torture or cruel, inhuman, or degrading treatment or punishment*. Laws or orders violating other established human rights are not addressed.

## Competence and conscience

The APA ethics code warns psychologists to "...take precautions to ensure that their potential biases, [and] the boundaries of their competence...do not condone unjust practices" (p. 3). In an overly optimistic stance on competence, the PENS Report states, "...special safeguards may be necessary to protect the rights and welfare of persons or communities whose vulnerabilities impair autonomous decision-making" [[6], p. 8]. Boundaries of competence are perhaps most severely tested in issues of religion and culture where, as the PENS Report mentions, "knowledge of race, ethnicity, culture, and national origin" is involved (p. 6). While there are no doubt efforts on the part of some U.S. military figures to become more culturally sensitive [71], there is overwhelming evidence that knowledge of culture has been abused to coerce information from detainees [16]. In settings where cultural knowledge has been abused, particularly capitalizing on sexual and religious taboos, even a mental health provider knowledgeable about cultural issues would find it difficult to utilize diversity education in a positive sense [68]. Furthermore, psychologists rarely possess sufficient knowledge about how other collaborators will use information that a psychologist identifies and shares. Even more challenging is the fact that the ethics code permits psychologists to work outside their areas of competence in emergencies. Unfortunately for the detainees, the military defines the "war-on-terror" – much as the PENS task force itself – as a perpetual emergency.

## The paucity of research and the high risk of error

Professional competency demands a research base, especially when work in such high stakes contexts is involved, and the lack of a good research base is a major reason to prohibit psychologist involvement in detainee interrogations. There is no established evidence that psychologists are the least bit helpful in ensuring ethical ways of eliciting information. There is no scientific literature that would lead us to conclude that psychologists in these interrogation settings provide unique competencies. We question the statement in the PENS report that U.S. national security interrogations have been "significantly developed and refined" [[6], p. 5]. We do not doubt that this work has expanded into more worldwide use, but dissemination itself does not portend positive growth.

Without empirical evidence that psychologists positively contribute to these interrogations – that their presence improves the veracity of the information obtained – then there is little reason to assume unique competencies. Meanwhile, social psychological research indicates that if anything, estimates of one's own competency can be seriously overestimated. No expert of any kind possesses error free methods of determining who is or who is not telling the truth in an interrogation, or who is and who is not a "terrorist." In fact forty years of research on the detection of deception shows there is a very high risk of error even among trained interviewers, and that deception detection accuracy is little better than chance [35]. The consequences of psychologist error in military detention sites could be severe, and perhaps most important, lead to punitive actions toward a detainee that is beyond the psychologist's control.

## Research ethics and detention center practice

It is a bitter irony that war-on-terror interrogations are so perilous for the research participant that no study resembling their conditions could be devised following the APA Ethics Code on Research. Internal Review Boards (IRBs) in the U.S., overseeing the protection of human participants, would never allow experiments in public or private universities that would approach the conditions in Guantanamo. IRBs and university researchers are sensitive to potential research abuse and afford research participants extensive protections [41,72]. Furthermore, without simulating high-risk degradation, no US study could provide the ecological validity to approach the conditions of "war-on-terror" interrogations. We have no doubt that to counter criticisms about *generalizability*, psychologists have considered secret research conducted within military or intelligence settings on detainees as research participants. We strongly object to such non-transparent research on the most vulnerable of populations [73]. However, since the APA has tied its interrogation policy to its ethics code, we hope that the APA would also find such research unacceptable.

Our optimism is tempered by awareness that, parallel to the changes to standard 1.02, clauses governing research were also weakened, providing greater leeway to dispense with *informed consent*, and permit the increased use of *deception*. Thus, standard 8.05 now allows informed consent to be disregarded "where otherwise permitted by law or federal or institutional regulations" [4]. Similarly, in a statement resembling the very definition of torture and the qualifying limits of "severe", clause 8.07 allows deception, except "Psychologists do not deceive prospective participants about research that is reasonably expected to cause physical pain or severe emotional distress" [4].

As all U.S. psychologists are accountable to all sections of the APA ethics code, it is worth noting that a psychologist consulting on interrogations is conducting an experiment of sorts. The justifiably strict codes for ethical research are therefore pertinent to these experiments. It is ironic in the sense that the PENS report is essentially encouraging very *real-world interrogation *practices that are substantially higher risk than *research in university settings*. In university settings, informed consent is required, deception carefully monitored, withdrawal from a study permitted without penalty, and debriefing essential [74,75].

Perhaps most ironic is the revolution that developed in social scientific ethics surrounding a series of studies on obedience conducted by Stanley Milgram [76]. This research examined the willingness of naive participants to inflict increasing levels of shock (to the point of implied death) on an ostensibly naïve participant (who was actually a planted, experimental confederate, and never harmed in the study).

Milgram's findings are relevant to the interrogation debate because they speak to psychologist-involvement in distant, secret special-operations detention centers under military command or at CIA black sites. The experiments depict the psychological pressures for obedience to authority active on human beings. These experiments additionally speak to the moral climate that once existed in the APA and the world of psychologists [47]. Those rigorous standards were designed to protect research participants (and researchers alike) from engaging, even inadvertently, in harmful research protocols; and producing even the temporary illusion in research participants that they were harming another human being.

It is obvious that the activities within Abu Ghraib, Guantanamo, CIA black sites, and U.S. Navy Brigs far exceed in intensity and duration the psychological harm to participants in the Milgram studies. After Milgram, the American Psychological Association worked to radically alter psychological research ethics. For this reason, Milgram's experiments were banned despite the hope that, with research knowledge and education, people could be inoculated against blind obedience [76]. It is worth noting that strong actions by the APA and ethics committee to address these concerns, at that time, increased public respect for and confidence in the profession.

## Knowledge derived from obedience studies

Meanwhile, we can hear the message of the Milgram and Zimbardo [77] experiments, that no single individual, regardless of education – whether a psychologist or non psychologist – is ever fully inoculated from the tendency to obey authority, especially within closed contexts. Work pressures in the detainee detention sites greatly amplify the dangerous situational factors. Interrogation psychologists are answerable to their commanding officer who has complete say in these special-ops. In these sequestered, deindividuating, government settings, authority figures are proximate and their needs and goals immediate, critical factors shown by Milgram to increase obedience [76].

Given the substantial physical and psychological distance of detention sites from the outside world (and the psychological distance of the interrogation team from the detainee), the CIA or U.S. military psychologist is in a position far more intense than any faced by Milgram's naïve participants [76]. Interrogators in these war-on-terror settings, pressed from above to get information, unable to differentiate between detainees who are lying and those who are telling the truth, are likely to become frustrated when faced with resistance [78].

Psychologists themselves are not inoculated from these vulnerabilities. Many are likely to invest more emotionally in the interrogation's success than did Milgram's participants. The Milgram experiments suggest that competence involves the ability to question and assess authority, to hold to one's perceptions and act accordingly. The authority of the Milgram experimenter pales in comparison to the authority of the commanding officer of a military operation. The authority is total and cannot be disobeyed without opening oneself to potentially severe consequences. Any psychologist at Guantanamo or the black sites who questions the orders of the commanding officer to get more intelligence with methods that offend the psychologist but not the commanding officer, could be severely disciplined.

## Conclusion and future directions

The stated aim of the PENS Report was to meet the "highest ideals" of the discipline. Indeed, the world's largest mental health organization should strive for nothing less. We have critiqued an array of flaws within the PENS Report, and yet we are optimistic that positive restorative steps will be taken. For instance, there is an opportunity now for the APA to provide U.S. psychologists, overseas and sequestered, with clear, firm, and unequivocal ethical guidelines. The APA can reaffirm its responsibility to protect its members, the field, and those impacted by its tools [1]. The APA can share in the responsibility for adverse actions caused by psychologists in foreign detention camps and CIA black sites. The APA can recognize that the legality of a setting is a necessary precondition of ethical practice. It can join the American Bar Association in recognizing the illegalities of these detention systems, confirming that national security concerns may be best met by embracing international law and *the Universal Declaration of Human Rights*.

The APA can also reframe its appropriation requests to the U.S. Congress. Rather than requesting additional money to study "effective" interrogation methods [79], the APA could attempt to bring increased attention and funding to their own largely ignored PENS statement that "...certain settings may instill in individuals a profound sense of powerlessness." (p. 6). The APA's lack of response to this understated truth reflects as much as anything the misshapen priorities of the whole PENS initiative. Whenever such profound "powerlessness" arises in any area of psychological practice, it should be responded to with a resounding call to reduce the sense of helplessness and resulting trauma. Psychologists should concentrate their efforts into healing and empowering individuals, not in exploiting a sense of powerlessness in the service of intelligence gathering. Fighting powerlessness and trauma should be a highest priority and should be addressed directly; it should never be mentioned, as in the PENS Report, as a mere afterthought.

The APA can ensure that its ethical standards hold as rigorously for illegally detained citizens of foreign nations as it does for U.S. clients in psychotherapy (and U.S. participants in research). We wish of course that the APA could have taken a more collaborative and humanitarian approach from the beginning, and that PENS had never come into existence. It is, however, never too late to direct resources toward a greater psychological understanding of other cultures, expanding communicative exchanges across all nations and religions [68,80]. Such a community-based psychological effort would have been an ideal substitute to a procrustean 'ethics of interrogation'. Yet there is little reason to wish for a better past with the opportunities offered by the present.

From the events of the PENS process, the APA can encourage research investigations into majority influence; invest in its own program evaluations to understand and prevent such group-think-based errors in the future. They can encourage the study of minority influence, whistle blowing, civil disobedience, and nonviolence when moral necessity calls.

Aggressive monitoring of psychologists in detention camps is another promising route for the APA, with a determined emphasize on accountability. The APA can publicly affirm that psychologist involvement violates APA ethics; demand an independent investigation of practices in detention sites to discover the truth about reports of abuse by psychologists; engage in restorative disciplinary action against psychologists who have already violated ethical standards; press the U.S. government to work collaboratively on stringent safeguards; and present itself as a protective authority for psychologists to turn to in morally complicated situations. If the APA believes itself ineffectual or unwilling to take on such tasks, an international group of psychologists (e.g. the psychological equivalent of the U.N.) should be developed that can seriously take on such organizational oversight.

The American Psychological Association has a long history of humanistic ideals, human rights, and the striving for a greater peace. During WWII, eminent psychologists from Gordon Allport to Edward Chase Tolman signed *the Psychologist's Manifesto *[81], calling on the utilization of psychological knowledge in the service of peace, opposing the abuse of power through psychological means. Psychologists have much to contribute to national security, world security, and world peace through nonviolence. The field possesses a rich potential to discover the negative consequences of verbal abuse, deprivation, dependence, and injustice. Let us follow the lead of our humanistic mentors with a greater passion. Let us increase respect for the discipline throughout the United States and across the world. There is much more to be accomplished for humanity. To feel good about our present effort, to deserve a positive acknowledgement from history, we are best off placing our actions in direct line with our ethics.

## Endnotes

a) Despite the very public connections between the task force and the ethics code, APA officials "observing" or guiding the task force insisted that no new ethical rules were to be considered during task force discussions. Perhaps the ethical rules were seen as threatening to positions held by those officials. Or perhaps there was a fear that changes in the ethics code would have to be made to support the report's conclusions, which might have delayed approval of the PENS Report. Either way, this task was left up to the APA Ethics Committee alone (Arrigo, J. M., personal communication, November 20, 2007).

b) Development of this same disorientation-dependence combination was, not long after Mr. al-Qahtani's interrogation, standard operating procedure in Guantanamo, as the explicit reason for the four weeks of isolation prescribed for all new detainees, designed with the intention of prepping the detainee for interrogation [20,82]. Furthermore, a few weeks after the standard operating procedures went into effect, medical and psychological monitoring was required during isolation by order of Defense Secretary Rumsfeld [83].

c) If Dr. Leso, the psychologist, was unable to detect the profound harm occurring to Mr. al-Qahtani, FBI agents at Guantanamo had no such difficulty. Over a month before this harsh interrogation ended, agents described Mr. al-Qahtani's condition: "In September or October of 2002 FBI agents observed that a canine was used in an aggressive manner to intimidate detainee __ after he had been subjected to intense isolation for over three months. During that time period, __ was totally isolated (with the exception of occasional interrogations) in a cell that was always flooded with light. By late November, the detainee was evidencing behavior consistent with extreme psychological trauma (talking to non-existent people, reporting hearing voices, crouching in the corner of a cell covered with a sheet for hours on end)" [84].

d) We are not here supporting the role of psychologists in behavioral drift prevention. We are rather clarifying the minimal conditions under which such a role can ethically be discussed. There are many other issues that argue against such a role for psychologists, including lack of competence, potential conflicts between their responsibilities to interrogators, to the military, and to the broader society, as well as the argument against abetting the illegal practices in U.S. detention centers that applies to all professionals in these facilities.

e) As of this writing, there has been no word on any action being taken in the case.

f) On numerous occasions, it has been announced by the APA that the PENS Report *had *to be based on extant Ethics Code provisions; otherwise it would have required a grueling year-long process to be adopted. Therefore, despite statements to the contrary, we fail to understand how such a similar process could be waived for the 2007 resolution. We hope, though, that our concern is unwarranted.

## Competing interests

The author(s) declare that they have no competing interests.

## Authors' contributions

Brad Olson wrote the first draft. Stephen Soldz and Martha Davis revised it thoroughly on multiple occasions. The final manuscript was the result of mutually agreed upon modifications by all authors.
